# Physicochemical Factors Affecting Microbiota Dynamics During Traditional Solid-State Fermentation of Chinese Strong-Flavor Baijiu

**DOI:** 10.3389/fmicb.2020.02090

**Published:** 2020-09-09

**Authors:** Tongwei Guan, Yijin Lin, Kebao Chen, Mengying Ou, Jiaxu Zhang

**Affiliations:** ^1^College of Food and Biological Engineering, Xihua University, Chengdu, China; ^2^Chengdu Shuzhiyuan of Liquor Co., Ltd., Chengdu, China

**Keywords:** solid-state fermentation, microbial succession, Baijiu, environmental factors, high-throughput sequencing

## Abstract

Spontaneous solid-state fermentation (SSF) of Chinese Baijiu involves diverse microbes from Daqu and pit mud (PM). Given that the transfer of interphase microflora during the fermentation is a continuous and dynamic process, longitudinal studies are essential to provide ecological insights into community stability and response to consecutive disturbances in the process. In this context, this study aimed to generate a comprehensive longitudinal characterization of the microbiota during the fermentation processes of Chinese strong-flavor Baijiu (CSFB) differing in cellar ages with consideration for potential relation to physicochemical variables. The microecology variations observed during the 6-years cellar SSF (SCSSF) and 30-years cellar SSF (TCSSF) processes reveal that fungal composition contributes to a larger extent than bacterial composition to such variations. Orders of *Lactobacillales*, *Anaerolineales*, *Enterobacteriales*, *Bacillales*, *Eurotiales*, and *Saccharomycetales* dominated (average relative abundances >10%) the microbiota in both SCSSF and TCSSF processes but with a different percentage in the operational taxonomic unit (out) abundances. Compared with the SCSSF process, TCSSF possessed slower microbial succession rates, which were in accordance with the profile of physicochemical properties. From a network perspective, the microbial community structure observed in the TCSSF processes was more stable than that in the SCSSF. This may benefit from the milder physicochemical conditions of the TCSSF processes, especially the temperature, which is also more beneficial to the growth of some groups that have negative effects on fermentation, such as *Staphylococcus*, *Pseudomonas*, and *Acinetobacter*.

## Introduction

With a long history, fermentation technology has been applied to impart desirable properties to food products, such as extended shelf life and organoleptic and nutritional properties (Smid and Hugenholtz, 2010; Smid and Lacroix, 2013). Most fermentation processes mentioned above are driven by mixtures of microbes originating from defined starters and processing environments (Bokulich and Mills, 2013). In addition to the defined starters, the attribution of variations in food quality to ambient microbiota is central to the current understanding of fermentation ecosystem function, particularly stability and safety (Bokulich et al., 2016; Wang et al., 2017b). Nevertheless, longitudinal studies are essential to provide ecological insights into community stability and response to consecutive disturbances (Faust et al., 2015).

Spontaneous solid-state fermentation (SSF), which relies heavily on environmental microorganisms, is extensively employed in the repeated-batch brewing process of Chinese strong-flavor Baijiu (CSFB) (Jin et al., 2017). This somehow empirical fermentation process is carried out in a rectangular cellar where the entire inner wall is covered with pit mud (PM) (Tao et al., 2014). In brief, fermentation grains (FGs), obtained from the last fermentation round are mixed evenly with crushed raw materials (sorghum, wheat, corn, rice, and sticky rice) for distilling. After distilling, the steamed mixture is supplied with 10–20% (w/w) Daqu, which mainly includes mold and yeast as starter cultures. Then, the above mixture is placed into the cellar and sealed with common mud for about 60–90 days’ anaerobic fermentation. After fermentation, the FG is taken out of the cellar and distilled to make Chinese liquor after supplying new raw materials, and then the process is repeated as described above (Tao et al., 2014).

In practice, it is widely believed that the flavor and quality of CSFB are closely correlated with PM quality, which is largely determined by PM microbial diversity and community structure (Liu et al., 2017). Microorganisms that colonize in the PM can migrate or diffuse into the FG, thereby affecting the composition and succession of primary communities (Ding et al., 2015; Wang et al., 2017a). Therefore, studying the PM microbiota is critical to understand the complete microbial ecosystem of Baijiu fermentation. Many studies have demonstrated that members of *Clostridia*, *Bacteroidia*, and *Methanobacteria* were regarded as core functional microorganisms in PM due to their capability to produce various organic acids such as lactate, butyric, and caproic acids (Ding et al., 2015; Zou et al., 2018). A recent diversity analysis across a quality gradient of PM revealed that the microbial composition of PM was significantly domesticated and shaped by environmental variables during the long-term production process (Hu et al., 2016). Unfortunately, despite the diversity and structure of PM microbiota that was extensively studied via cross-sectional analyses, the transfer of interphase microflora during the fermentation is a continuous and dynamic process, and its extent of influence on primary communities is still unclear (Wang et al., 2017a).

On the other hand, operating parameters might also affect the microbial community structure and succession, which in turn will affect the microbiota’s performance during the fermentation process (Jiang et al., 2016; Tan et al., 2019). For instance, Li P. et al. (2016) revealed that the operating temperature plays a critical role in defining the functional population residing in the Daqu. However, so far, efforts targeting the relationship between microbial composition and physicochemical conditions during the SSF of CSFB, such as temperature, acidity, and pH, are relatively scarce.

In this context, this study aimed to generate a comprehensive longitudinal characterization of the microbiota during the fermentation processes of CSFB, considering cellar age as the determinant of the fermentation system. Accordingly, the effect of cellar age on population dynamics during the fermentation was examined. For this purpose, two fermentation processes were started up under similar operating conditions but different cellar ages (6 and 30 years). The microbial community structure and dynamics of FG were monitored using Illumina HiSeq sequencing technology, and the results were correlated with physicochemical conditions.

## Materials and Methods

### Experimental Design and Sampling

The study was conducted at a famous CSFB distillery in Sichuan province, China (30°, 38′N; 103°, 42′E), with an average annual temperature of 18°C. We selected two different batches of cellars (cellar-S and cellar-T), which were used uninterruptedly for 6 and 30 years, respectively. FG samples were separately collected on days 0, 7, 15, 25, 45, 70, and 95 according to temperature evolution during the SSF process (Tan et al., 2019). To obtain adequate information and representation, samples from each time point were composed of six subsamples collected from different spatial positions of the cellar (200 g per subsample) (Supplementary Figure S1). Three parallel samples (three random cellars) were collected for each sample type. Finally, the 42 samples were transported into the lab on ice and stored at −80°C until analyzed.

### Physicochemical Properties of Samples

The core temperature of sampling sites was continuously monitored using a probe thermograph (Huahai WSS-481, Jiangsu, China) inserted into the center of the cellar. Moisture content was determined by the dry/wet weight method at 105°C. The pH level was measured in the suspension (10 g samples suspended in 50 ml ultrapure water) by a pH meter (Thermo Fisher Scientific, Waltham, MA, United States). The total titratable acidity was determined by titration with 0.02 M NaOH yielding a titration endpoint of pH 8.2. Samples (10 g) were suspended with 90 ml ultrapure water, ultrasonically treated at 0°C for 30 min, and then centrifuged at 4°C for 5 min; the obtained supernatant was used to determine the content of glucose (the DNS method) (Miller, 1959; Bravo et al., 1998). The starch content of FGs was calculated by calculating the difference between total reducing sugar and the original reducing sugar after hydrolysis with HCl (20%, v/v) for 30 min. The ethanol content was determined via alcoholmeter after distillation (Tan et al., 2019). All physicochemical analyses were conducted in triplicate.

### Metagenomic DNA Extraction, Amplification, and Sequencing

Metagenomic DNA was extracted from 200 mg of each homogenized and freeze-dried FG samples using the Power Soil^®^ DNA Isolation Kit (MO Bio Laboratories, Carlsbad, CA, United States) according to the manufacturer’s protocol. The presence and quantity of genomic DNA were checked with a NanoDrop ND-1000 spectrophotometer (Thermo Fisher Scientific, Dreieich, Germany), and the extracted DNA was diluted to 10 ng/μl as a template for further amplification.

The V3–V4 hypervariable region of bacterial 16S rRNA genes was amplified with the universal primer pair 338F (5′-ACTCCTACGGGAGGCAGCA-3′) and 806R (5′-GGACTACHVGGGTWTCTAAT-3′) with the barcodes (Caporaso et al., 2010). PCRs were carried out with 30 cycles of a 25 μl reaction volume containing 5 ng DNA templates, 1 U of EX Taq (Takara, Otsu, Japan), 1 × Ex Taq buffer, 0.2 mM of each dNTP, and 0.4 μM of each primer. The thermal cycle profile after an initial 2 min denaturation at 98°C was as follows: denaturation at 98°C for 30 s, annealing at 50°C for 30 s, and extension at 72°C for 10 s with the final extension at the same temperature for 5 min. For fungi, the primer pair ITS1F (5′-GCTGCGTTCTTCATCGATGC-3′) and ITS2 (5′-GCTGCGTTCTTCATCGATGC-3′) targeting the ITS1 regions was adopted to analyze fungal taxa (Huang et al., 2016). The PCR (25 μl) consisted of 10 ng of DNA templates, 1 U of EX Taq (TaKaRa, Dalian, China), 1 × Ex Taq buffer, 0.2 mM of each dNTP, and 0.4 μM of each primer. Thermal cycling consisted of initial activation at 95°C for 5 min, followed by 30 cycles of denaturation at 95°C for 30 s, annealing at 50°C for 30 s, and elongation at 72°C for 40 s and finally elongation at 72°C for 7 min. Negative controls were treated similarly with the exclusion of template DNA.

The amplicons were purified with a PCR purification kit (TaKaRa, Dalian, China), after which an Illumina TruSeq DNA sample preparation LT kit (San Diego, CA, United States) was used to construct the sequencing libraries, before sequencing using the Illumina HiSeq 2500 platform with a 2 × 250 bp paired-end protocol (Illumina, San Diego, CA, United States).

### Bioinformatic and Statistical Analysis

The paired-end DNA fragments were initially assembled via FLASH (Zhang et al., 2014). Subsequently, the raw sequences were de-multiplexed, trimmed, and assessed for chimeras using QIIME software (v. 1.8.0) (Caporaso et al., 2010). Quality-filtered sequences were then clustered into operational taxonomic units (OTUs) using UCLUST software with a minimum confidence threshold of 0.97 (Edgar, 2010). The representative sequences from each clustered OTU were taxonomically assigned using the Silva database (16S rDNA) and Unite database (ITS) in QIIME (Caporaso et al., 2010). It is likely that chloroplast and chondriosome sequences were removed from further downstream analysis. Alpha diversity indices were calculated via QIIME after normalizing sequences to the lowest sequence number (32,654 reads for bacteria and 59,856 reads for fungi, respectively).

Significant differences in alpha diversity metrics were tested using one-way analysis of variance (ANOVA) with Duncan’s test. Variabilities of microbial assemblage structure among two batches were determined though principal coordinate analysis (PCoA) based on Bray–Curtis dissimilarities (Vázquez et al., 2013) and tested for significance with ANOSIM (Clarke, 1993). Distance-based redundancy analysis (db-RDA) between microbial communities and continuous variables was performed via the vegan package in R^1^. Spearman’s rank correlations between genera were calculated to reveal the relationships among microbial communities. Only significant correlations (*p* < 0.05, with false discovery rate correction) were considered as valid correlations. Network was created via Gephi (Web Atlas, Paris, France) to sort through and visualize the correlations (Bastian et al., 2009).

## Results

### Physicochemical Properties

**FIGURE 1:**
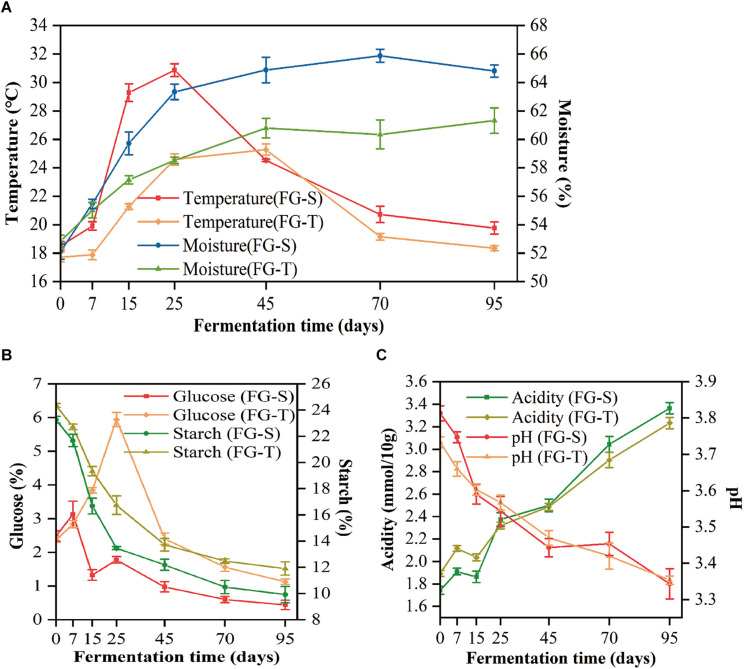
Dynamics of physicochemical characteristics during the SCSSF and TCSSF processes. **(A)** Changes in core temperature and moisture of fermentation grains. **(B)** Changes in reducing sugar (calculated as glucose) and starch contents. **(C)** Changes in pH and titratable acidity.

The physicochemical properties of FGs characterized the process of CSFB fermentation. Thereinto, the temperature profiles of the TCSSF processes lagged behind those of SCSSF processes, which were in accordance with the profiles of glucose and starch consumptions (Table 1). Specifically, the temperature of SCSSF processes increased markedly from 18.51 to 29.28°C on days 0 to 15, while obviously smaller increments of TCSSF processes from 17.72 to 21.17°C were observed, and the top temperature of SCSSF processes observed on day 25 was 5.58°C higher than that of TCSSF processes observed on day 45. The moisture increased steadily from 52.82 to 58.52% and from 52.11 to 63.34% during the TCSSF and SCSSF processes on days 0 to 25 and remained around 60.81 and 65.18% until the end of fermentation, respectively (Figure 1A). The glucose contents showed a substantially increasing tendency during the TCSSF processes on days 0–25 (2.37 to 5.94%), followed by a quick depletion on days 25–45 (5.94 to 2.41%). Conversely, a noteworthy decrease of glucose contents could be observed during the SCSSF processes on days 7–15 (3.13 to 1.78%). Moreover, the starch contents decreased from 24.36 to 16.74% and from 23.26 to 13.46% during the TCSSF and SCSSF processes on days 0 to 25 and finally fell to 11.91 and 9.92%, respectively (Figure 1B). Similar evolution profiles of titratable acidity and pH values were observed during the SCSSF and TCSSF. The titratable acidity increased from 1.74 to 3.36 mmol/10 g and from 1.89 to 3.23 mmol/10 g, and the pH values decreased from 3.81 to 3.34 and from 3.73 to 3.35, respectively (Figure 1C).

**TABLE 1:** Chemical properties of FG during fermentation^*a*^.

**Sample ID**	**Temperature (°C)**	**Moisture (%)**	**Acidity (mmol/10 g)**	**pH**	**Glucose (%)**	**Starch (%)**	**Ethanol (%)**
S0	18.51 ± 0.31	52.11 ± 0.53	1.74 ± 0.04	3.81 ± 0.02^a^	2.48 ± 0.16^e^	23.26 ± 0.26^b^	0
S7	19.92 ± 0.30	55.47 ± 0.33	1.91 ± 0.03	3.75 ± 0.02^b^	3.13 ± 0.40^c^	21.67 ± 0.47^c^	0.32 ± 0.08
S15	29.28 ± 0.62^b^	59.72 ± 0.79^d^	1.86 ± 0.05	3.59 ± 0.03^d^	1.33 ± 0.16	16.68 ± 0.59^e^	3.04 ± 0.25
S25	30.86 ± 0.45^a^	63.34 ± 0.54^b^	2.37 ± 0.06	3.54 ± 0.04^e^	1.78 ± 0.09	13.46 ± 0.11	6.45 ± 0.32^a^
S45	24.52 ± 0.08^d^	64.87 ± 0.89^a^	2.50 ± 0.06^e^	3.44 ± 0.03	0.98 ± 0.15	12.20 ± 0.42	5.83 ± 0.56^b^
S70	20.72 ± 0.57^e^	65.87 ± 0.46^a^	3.04 ± 0.07^c^	3.45 ± 0.03	0.60 ± 0.09	10.49 ± 0.51	5.59 ± 0.40^c^
S95	19.77 ± 0.43	64.80 ± 0.43^a^	3.36 ± 0.05^a^	3.34 ± 0.04	0.44 ± 0.14	9.92 ± 0.62	4.96 ± 0.24^d^
T0	17.72 ± 0.35	52.82 ± 0.44	1.89 ± 0.03	3.73 ± 0.02^b^	2.37 ± 0.08^e^	24.36 ± 0.15^a^	0
T7	17.88 ± 0.35	55.02 ± 0.54	2.12 ± 0.03	3.66 ± 0.02^c^	2.84 ± 0.10^d^	22.72 ± 0.22^b^	0.1 ± 0.02
T15	21.27 ± 0.20^e^	57.16 ± 0.29	2.04 ± 0.03	3.60 ± 0.02^d^	3.84 ± 0.08^b^	19.33 ± 0.36^d^	1.85 ± 0.26
T25	24.58 ± 0.39^d^	58.52 ± 0.23^e^	2.32 ± 0.03	3.57 ± 0.02^d,e^	5.94 ± 0.21^a^	16.74 ± 0.71^e^	4.45 ± 0.35^e^
T45	25.28 ± 0.39^c^	60.78 ± 0.69^c,d^	2.49 ± 0.03^e^	3.47 ± 0.02	2.41 ± 0.17^e^	13.73 ± 0.49	5.34 ± 0.65^c,d^
T70	19.15 ± 0.24	60.34 ± 1.02^c,d^	2.90 ± 0.07^d^	3.42 ± 0.04	1.56 ± 0.13	12.48 ± 0.17	4.76 ± 0.42^d,e^
T95	18.35 ± 0.17	61.32 ± 0.90^c^	3.23 ± 0.05^b^	3.35 ± 0.01	1.13 ± 0.08	11.91 ± 0.50	4.45 ± 0.24^e^

*All data are presented as means ± standard deviations (*n* = 3). Values with different letters in a column mean significant differences at *p* < 0.05 as determined by one-way ANOVA Duncan’s test.*

### Microbial Composition and Dynamics

Microbial composition and dynamics in the SCSSF and TCSSF processes were identified via Illumina HiSeq sequencing. After filtering the low-quality reads and chimeras, 3,320,616 bacterial and 4,365,089 fungal effective tags were obtained from all samples, with an average length of 424 and 262 bp, respectively. Rarefaction analyses indicated that the microbial communities were well represented, as the rarefaction curves approached saturation plateau (Supplementary Figure S2).

**FIGURE 2:**
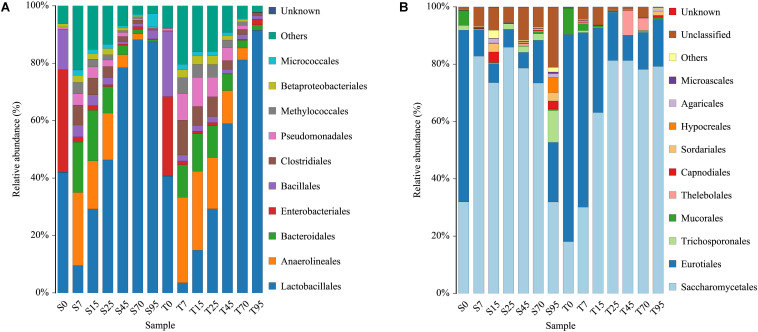
Dynamics of relative abundances of the major bacterial **(A)** and fungal **(B)** orders during the SCSSF and TCSSF processes, as obtained by Illumina HiSeq sequencing analysis.

After filtering the database with a 0.005% relative abundance filter, the OTUs were annotated and classified at order levels. In general, highly similar trends of population dynamics were observed during the SCSSF and TCSSF processes but with a different percentage in the OTU abundances (Figure 2). Orders of *Lactobacillales*, *Enterobacteriales*, *Bacillales*, *Eurotiales*, and *Saccharomycetales* dominated (average relative abundances >10%) the initial FG community in both SCSSF and TCSSF processes. Among these prevailing orders, (i) the relative abundance of *Lactobacillales* decreased markedly during the SCSSF and TCSSF processes from 42.02 to 9.52% and 40.65 to 3.53% on days 0 to 7, then increased gradually, and represented the largest fraction in range of 46.33–88.19% and 29.29–91.46% on days 25–95, respectively; (ii) *Enterobacteriales* and *Bacillales* shifted to subdominant orders (1%< average relative abundances <10%) during the first 7 days of fermentation, with the relative abundance decreased from 35.66 to 1.84% and 13.88 to 4.11% for SCSSF processes, whereas it decreased from 27.61 to 1.31% and 22.74 to 2.07% for TCSSF processes, respectively; (iii) the relative abundance of *Saccharomycetales* in SCSSF processes increased substantially from 31.92 to 82.81% on days 0 to 7, whereas in TCSSF processes, it increased gradually from 18.06 to 81.29% on days 1 to 25 and then dominated until the end of SCSSF and TCSSF processes, and almost opposite dynamics of *Eurotiales* were observed. The microbial communities of SCSSF and TCSSF processes were also co-dominated by *Anaerolineales* and *Bacteroidales* on day 7, and these two orders maintained their high abundances until day 25. In contrast, *Trichosporonales* co-dominated only in the end of SCSSF processes. *Mucorales* was regarded as a subdominant order in SCSSF processes on day 0 and in TCSSF processes on days 0–7.

At the genus level, only the genus that appeared twice in one sample type was considered as a valid genus. In total, 365 bacterial and 60 fungal genera were obtained from all samples. The genus-level classification of the bacterial community showed that the initial FG communities in both SCSSF and TCSSF processes were mainly dominated by strict aerobes (e.g., *Staphylococcus* and *Acetobacter*) or facultative aerobes (e.g., *Kosakonia*, *Lactobacillus*, *Weissella*, *Pediococcus*, *Pantoea*, and *Bacillus*), and they took up over 86% of the total abundance (Supplementary Table S1). Thereinto, *Weissella* (17.40 vs 12.05%), *Staphylococcus* (8.76 vs 5.95%), and *Bacillus* (13.71 vs 6.94%) were more abundant in TCSSF processes than in SCSSF. During the first 7 days of fermentation, except for *Acetobacter*, the relative abundance of these strict or facultative aerobes was reduced substantially, while *Acinetobacter*, *Pseudomonas*, *Methylobacter*, *Longilinea*, and *Clostridium* increased markedly. Afterward, *Lactobacillus* increased gradually and represented the largest fraction as the fermentation went on.

The fungal community was dominated by the genus *Kazachstania* with a significant level of *Thermoascus*, *Aspergillus*, and *Saccharomyces* and minor presence of *Thermomyces*. At the beginning of fermentation, the FG community in both SCSSF and TCSSF processes was dominated by *Thermoascus* (38.35%) and *Aspergillus* (33.14%). As the fermentation went on, the *Thermoascus* and *Aspergillus* presence decreased with a concomitant increase in *Kazachstania* (0.02–79.71%) and *Saccharomyces* (0–23.82%) abundance. It is striking to find that there were significantly more *Saccharomyces* and low *Aspergillus* taxa present in SCSSF processes than in TCSSF processes (*p* < 0.05) (Supplementary Table S1).

### Microbial Richness and Diversity

Alpha diversity indexes were conducted to evaluate the microbial richness and diversity that varied during the SCSSF and TCSSF processes (Table 2). The microbial richness (OTUs and Chao1) and diversity (Shannon) indexes were observed to increase substantially in the prophase (days 0–7) of the SCSSF and TCSSF processes and then declined modestly as the fermentation went on. For bacterial communities, the evolution trends of alpha diversity indexes in SCSSF processes were markedly more radical than those in TCSSF processes. For instance, the observed OTU indexes during the SCSSF processes increased substantially from 132 to 668 on days 0 to 7, whereas during TCSSF processes, they increased gradually from 144 to 595 on days 1 to 25 and afterward decreased. The evolution patterns of Chao1 and Shannon values were consistent with the OTU index. In contrast, for fungal communities, moderate upward trends of richness indexes were observed during the SCSSF processes compared with TCSSF processes.

**TABLE 2:** Summary of diversity index for microbial communities based on 16S rRNA and ITS1 gene sequencing data (cutoff = 0.005).

**Gene**	**Sample group**	**Sample ID**	**Clean reads**	**Alpha diversity metrics**		
				**OTU number**	**Chao1**	**Shannon**
16S rRNA gene	Fermented grains from 6-years cellars	S0	85,411.33 ± 24,774.82^a^	132.667 ± 5.508^e^	161.928 ± 9.316^de^	2.955 ± 0.095^c^
		S7	88,674.00 ± 25,556.67^a^	668.000 ± 9.539^a^	703.017 ± 25.707^a^	4.645 ± 0.144^a^
		S15	86,759.00 ± 11,543.91^a^	650.667 ± 17.786^a^	683.005 ± 16.981^a^	4.209 ± 0.571^a^
		S25	77,252.67 ± 7,914.13^a^	584.333 ± 75.659^ab^	654.738 ± 62.682^a^	3.069 ± 1.230^b,c^
		S45	72,487.67 ± 17,313.82^a^	479.667 ± 19.731^c,d^	586.012 ± 43.211^a,b^	1.631 ± 0.527^d,e^
		S70	79,761.00 ± 20,257.24^a^	411.333 ± 76.159^d^	529.301 ± 69.223^b,c^	0.964 ± 0.355^e^
		S95	61,048.33 ± 21,817.11^a^	173.667 ± 11.060^e^	221.071 ± 38.751^d^	0.928 ± 0.341^e^
	Fermented grains from 30-years cellars	T0	77,209.00 ± 20,652.86^a^	144.000 ± 6.083^e^	179.667 ± 16.966^d,e^	2.753 ± 0.114^c^
		T7	73,004.67 ± 14,485.12^a^	523.667 ± 116.663^b,c^	640.004 ± 14.256^a,b^	4.057 ± 0.371^a,b^
		T15	85,475.00 ± 11,085.05^a^	595.000 ± 17.088^a,b^	640.912 ± 19.105^a,b^	4.117 ± 0.535^a,b^
		T25	75,747.00 ± 20,257.17^a^	589.000 ± 60.108^a –c^	669.686 ± 34.141^a^	4.143 ± 0.858^a,b^
		T45	84,497.00 ± 21,211.93^a^	523.000 ± 42.462^b,c^	600.857 ± 48.286^a^	2.477 ± 0.568^c,d^
		T70	85,070.33 ± 33,460.74^a^	381.667 ± 142.759^d^	449.071 ± 187.814^c^	1.328 ± 0.262^e^
		T95	50,453.67 ± 24,837.44^a^	88.000 ± 15.716^e^	107.079 ± 4.423^e^	0.793 ± 1.025^e^
ITS1 gene	Fermented grains from 6-years cellars	S0	127,777.30 ± 29,252.08^a^	62.000 ± 5.292^f^	69.109 ± 6.891^d^	1.562 ± 0.118^c –f^
		S7	96,689.00 ± 21,542.83^a,b^	95.000 ± 7.211^a –d^	106.042 ± 12.237^a –c^	1.577 ± 0.143^c –f^
		S15	99,241.67 ± 14,200.82^a,b^	116.000 ± 12.124^a^	128.607 ± 5.380^a^	2.129 ± 0.758^b,c^
		S25	93,188.33 ± 4,787.20^a,b^	101.000 ± 9.000^a –c^	113.810 ± 17.829^a,b^	1.834 ± 0.217b^c –f^
		S45	110,710.00 ± 11,894.14^a,b^	98.333 ± 15.373^a –c^	101.044 ± 15.399^a –c^	2.117 ± 0.083^b,c^
		S70	96,658.67 ± 22,447.64^a,b^	106.667 ± 4.509^b –e^	108.709 ± 4.652^a –c^	2.356 ± 0.147^b^
		S95	84,384.67 ± 34,498.28^b^	96.667 ± 16.196^a –c^	102.700 ± 20.429^a –c^	2.858 ± 0.339^a^
	Fermented grains from 30-years cellars	T0	132,562.00 ± 12,909.96^a^	68.000 ± 5.196^e,f^	82.423 ± 4.759^c,d^	1.521 ± 0.078^d –f^
		T7	79,332.33 ± 11,985.76^b^	109.333 ± 1.528^a,b^	115.365 ± 6.281^a,b^	2.045 ± 0.153b^c,d^
		T15	134,318.30 ± 12,019.16^a^	88.667 ± 18.452^b –e^	97.225 ± 27.627^b –d^	1.872 ± 0.137^b –e^
		T25	106,741.00 ± 26,865.53^a,b^	87.667 ± 18.771^b –e^	103.988 ± 24.452^a –c^	1.262 ± 0.220^f^
		T45	109,846.30 ± 23,501.40^a,b^	84.000 ± 5.568^c –e^	93.302 ± 6.918b^c,d^	1.308 ± 0.156^e,f^
		T70	98,865.67 ± 29,523.02^a,b^	88.667 ± 8.505^b –d^	100.917 ± 16.962^a –c^	1.701 ± 0.563^c –f^
		T95	84,582.67 ± 26,941.50^b^	73.333 ± 17.616^d –f^	81.423 ± 20.529^c,d^	1.489 ± 0.133^d –f^

*All data are presented as means ± standard deviations (*n* = 3). Values with different letters in a column mean significant differences at *p* < 0.05 as determined by one-way ANOVA Duncan’s test.*

**FIGURE 3:**
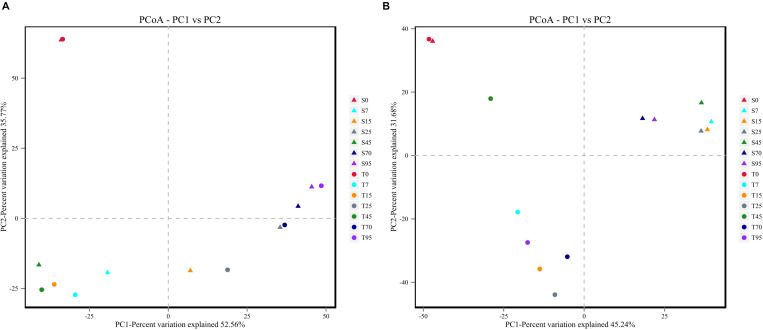
The Bray–Curtis distance PCoA of bacterial **(A)** and fungal **(B)** communities during the SCSSF and TCSSF processes, as obtained by Illumina HiSeq sequencing analysis.

In addition, PCoA plots based on Bray–Curtis dissimilarity were generated to compare the bacterial and fungal communities in the processes. Similar evolution patterns of the bacterial community were observed during the SCSSF and TCSSF processes (Figure 3A). On the contrary, for fungal communities, samples from SCSSF and TCSSF processes were clearly separated in the PCoA ordination space (Figure 3B). Moreover, ANOSIM tests indicated that high similarities (*R* = −0.048, *p* = 0.636) in bacterial communities but significant differences (*R* = 0.499, *p* = 0.002) in fungal communities were displayed in the SCSSF and TCSSF processes. Nevertheless, the initial samples from SCSSF (S0) and TCSSF (T0) processes were expectedly clustered into a cluster based on the dissimilarity distances of both bacterial and fungal communities.

### Relationships Between Environment Variables and Microbial Communities

**FIGURE 4:**
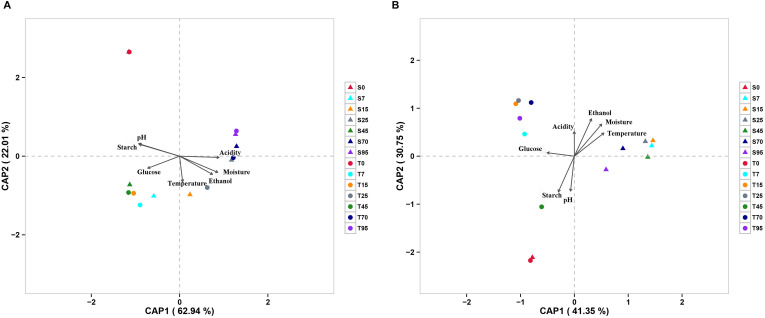
Distance-based redundancy analysis (db-RDA) of bacterial **(A)** and fungal **(B)** community structures as obtained by Illumina HiSeq sequencing and measurable environmental variables.

The effect of continuous variables on the bacterial and fungal community distributions was depicted by db-RDA (Figure 4). Starch and pH showed positive correlations with bacterial and fungal compositions at the startup stages (days 0–7), which was opposite to moisture, acidity, and ethanol. Glucose was strongly positively related to bacterial and fungal compositions at the heating stages (days 7–25) but negatively related to bacterial and fungal compositions at the cooling stages (days 25–95). Furthermore, Mantel tests showed that changes in starch, moisture, and pH had significant effects on bacterial composition in both SCSSF and TCSSF processes. Starch and moisture only had significant effects on fungi composition during the TCSSF process (Table 3).

**TABLE 3:** Mantel test correlation coefficient (*r*) and *p*-value for bacterial and fungal communities and measurable environmental variables during the SCSSF (cellar-S) and TCSSF processes (cellar-T).

**Classification**	**Sample group**	**Temperature**	**Moisture**	**Acidity**	**pH**	**Glucose**	**Starch**
Bacteria	Cellar-S	*r* = −0.039, *p* = 0.460	*r* = 0.850, *p* = 0.001	*r* = 0.457, *p* = 0.038	*r* = 0.784, *p* = 0.002	*r* = 0.573, *p* = 0.016	*r* = 0.807, *p* = 0.001
	Cellar-T	*r* = −0.154, *p* = 0.650	*r* = 0.707, *p* = 0.003	*r* = 0.559, *p* = 0.014	*r* = 0.763, *p* = 0.002	*r* = 0.164, *p* = 0.186	*r* = 0.673, *p* = 0.003
Fungi	Cellar-S	*r* = −0.056, *p* = 0.506	*r* = 0.474, *p* = 0.086	*r* = 0.278, *p* = 0.165	*r* = 0.565, *p* = 0.030	*r* = 0.163, *p* = 0.235	*r* = 0.421, *p* = 0.052
	Cellar-T	*r* = 0.146, *p* = 0.157	*r* = 0.882, *p* = 0.001	*r* = 0.189, *p* = 0.223	*r* = 0.595, *p* = 0.016	*r* = −0.344, *p* = 0.997	*r* = 0.789, *p* = 0.001

In addition, a statistical analysis based on Pearson correlation was run to evaluate significant correlations between identified taxonomic groups and environment variables. A significant positive/negative correlation (*p* < 0.05) was found between most major genera (average relative abundance >0.5) and the various environment parameters. However, *Kazachstania*, *Flavobacterium*, and *Fermentimonas* were significantly correlated only to temperature (Table 4).

**TABLE 4:** Pearson correlation coefficient (*r*) and *p*-value for identified bacterial and fungal orders/genera (average relative abundance >0.5%).

**Order/genus**	**Correlation with**	***p*-value**	***r***
Bacteria			
*Lactobacillales*	Moisture, Acidity, pH, Glucose, Starch	0.00, 0.00, 0.00, 0.00, 0.00	0.71, 0.85, −0.79, −0.70, −0.82
*Lactobacillus*	Moisture, Acidity, pH, Glucose, Starch, Alcohol	0.00, 0.00, 0.00, 0.01, 0.00, 0.00	0.83, 0.91, −0.90, 0.67, −0.92, 0.81
*Weissella*	Moisture, pH, Starch, Alcohol	0.01, 0.01, 0.02	−0.68, 065, 0.67, −0.63
*Pediococcus*	Moisture, Acidity, pH, Alcohol	0.00, 0.02, 0.00, 0.01	−0.77, −0.60, 0.77, −0.67
*Bacillales*	Moisture, pH, Starch	0.01, 0.01, 0.01	−0.67, 0.64, 0.66
*Bacillus*	Moisture, Acidity, pH, Starch, Alcohol	0.01, 0.04, 0.01, 0.01, 0.01	−0.63, −0.56, 0.68, 0.69, −0.63
*Staphylococcus*	Moisture, pH, Alcohol	0.02, 0.04, 0.03	−0.60, 0.56, −0.56
*Rhodospirillales*			
*Acetobacter*	Acidity, pH, Starch, Alcohol	0.04, 0.00, 0.01, 0.01	0.73, −0.54, 0.67, −0.67
*Enterobacterales*	Moisture, pH, Starch	0.01, 0.01, 0.02	−0.68. 0.66, 0.63
*Enterobacter*	Starch, Alcohol	0.01, 0.02	0.66, −0.64
*Pantoea*	pH, Starch, Alcohol	0.02, 0.04, 0.05	0.63, 0.55, −0.54
*Bacteroidales*			
*Fermentimonas*	Temperature	0.05	0.54
*Flavobacteriales*			
*Flavobacterium*	Temperature	0.00	0.721
*Fungi*			
*Eurotiales*	Temperature, Moisture, pH, Starch	0.02, 0.00, 0.02, 0.00	−0.62, −0.75, 0.60, 0.75
*Aspergillus*	Temperature, Moisture, pH, Starch, Alcohol	0.02, 0.00, 0.05, 0.00, 0.00	−0.59, −0.72, −0.54, 0.73, −0.73
*Thermoascus*	Moisture, Acidity, pH, Alcohol	0.00, 0.04, 0.00, 0.00	−0.77, −0.55, 0.73, −0.75
*Saccharomycetales*	Temperature	0.03	0.50
*Kazachstania*	Temperature	0.05	0.54
*Saccharomyces*	Moisture, Alcohol	0.01, 0.05	0.65, 0.54
*Mucorales*	Moisture, pH, Starch	0.01, 0.03, 0.01	−0.66, 0.59, 0.66
*Rhizomucor*	Moisture, Starch, Alcohol	0.02, 0.02, 0.02	−0.62, 0.61, −0.61

*Only correlations with *p* ≤ 0.05 are shown.*

### Interaction Networks of Microbial Communities in FG

**FIGURE 5:**
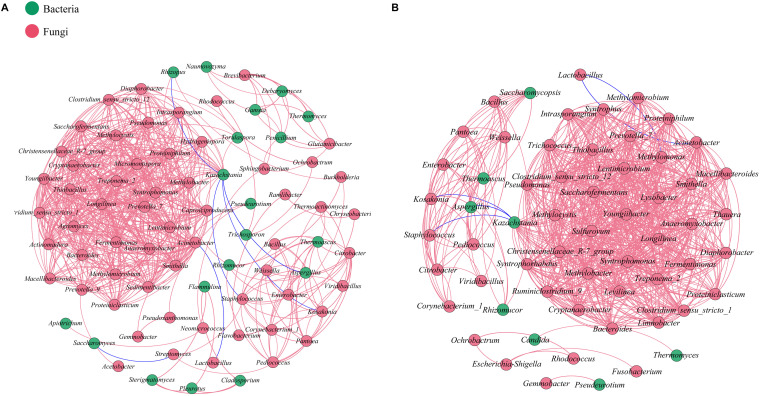
Relationships among microbial communities of SCSSF **(A)** and TCSSF **(B)** processes. A connection stands for a significant (*p* < 0.05) and strong (Spearman’s | ρ| >0.7) correlation. The color of each node is proportional to the types of microorganisms. Bacteria, red; fungi, green.

Network analysis based on Spearman’s rank correlation (| ρ| >0.7 and *p* < 0.05) was conducted to evaluate interactions among microbiota at the genus taxonomic level in each batch (Figure 5). In total, 627 pairs of significant and robust relationships from 78 genera and 708 pairs from 60 genera were identified during the SCSSF and TCSSF processes, separately. Compared with the SCSSF-specific network, the TCSSF had higher weighted degree (23 vs 16), graph density (0.4 vs 0.21), and average clustering coefficient (0.92 vs 0.86), suggesting more network hubs and closer connection of network modules.

## Discussion

Spontaneous SSF of Chinese Baijiu was triggered and mediated to a large extent by microorganisms that colonize in the PM (Jin et al., 2017). Currently, the characterization of the microbial communities of PM and FG is proliferating with the introduction of next-generation high-throughput sequencing technology (Sogin et al., 2006; Ding et al., 2015). However, restricted to obtaining samples from the closed fermentation process, efforts targeting the relationship between microbial composition and environmental conditions, such as temperature, acidity, and pH, are relatively scarce. This study aimed to generate a comprehensive longitudinal characterization of the microbiota during the fermentation processes of CSFB differing in cellar ages with consideration for potential relation to environmental variables.

In general, similar trends of physicochemical variables were observed during the SCSSF and TCSSF processes and followed the quintessential evolution of CSFB SSF processes (Tan et al., 2019). Thereinto, the temperature was increased substantially on days 0–25, which can be attributed to the flourishing growth of the microbial community that generated a large amount of bio-heat (Troy et al., 2012; Xiao et al., 2017). Furthermore, the temperature profiles of the TCSSF processes lagged behind those of SCSSF processes, which were in accordance with the profiles of bacterial and fungal richness (Chao1). It implies that the operating temperature plays a critical role in characterizing and defining the microbial succession (Ghanimeh et al., 2013). As expected, a rapid increase of glucose contents was observed at the heating stage with a concomitant decrease in starch contents, highlighting the intense microbial decomposition activity (Tan et al., 2019). The increase in titratable acidity throughout the process was estimated to be driven by the proliferation of positively related bacteria, such as the genera *Lactobacillus* and *Acetobacter*, the major contributors to lactic acid and acetic acid, respectively (Jung et al., 2012; Li S. et al., 2016). As a consequence, the acidic stress inhibited the growth of acid-sensitive microorganisms, such as the genus *Bacillus* (Tang et al., 2011). Note that a downward fluctuation in titratable acidity was observed on days 7–15, which may be due to yeasts using organic acids as an alternative carbon source under the aerobic and glucose-deficient conditions (Lu et al., 2004; Vermeulen et al., 2012). Given the micro-aerobic conditions structured by the large number of voids in FG, this situation was likely to occur (Ding et al., 2015). The moisture might affect the microbial community structure and succession (Bal et al., 2017), which in turn will affect the yield and quality of based liquors (Tan et al., 2019). In this study, a significant correlation was found between moisture and numerous bacteria and fungi at the heating stage. And it should be noted that the moisture remained at a relatively stable level at the later stage, which was possible due to the low biological activity characterized by decreased temperature and microbial diversity, suggesting that the moisture characterized the stage shift of fermentation. Furthermore, the determination of chemical indices may be influenced by the moisture without correction (Zhang et al., 2020).

Although a highly similar microbiome, presumably due to the identical inoculum, was observed in the initial samples of two processes, the bacterial and fungal communities rapidly underwent strong microbiome restructuring. The step change in bacterial community at the start-up stage (days 0–7) was mainly characterized by decreased relative abundances of *Lactobacillales* and *Enterobacteriales*, which were the dominant order in the initial community and replaced by *Anaerolineales*, *Bacteroidales*, and *Clostridiales*, among other prevailing orders. Order *Anaerolineales* was represented by *Anaerolineaceae*, whose all authentic species have been distributed widely in various types of anaerobic ecosystems, such as the subsurface, sediments, hot springs, and sludge, suggesting their ubiquity and function significance in such environments (Yamada et al., 2007). In this study, the presence of dominant *Anaerolineales*, with a concomitant decrease in strict (e.g., *Staphylococcus* and *Acetobacter*) and facultative aerobes (e.g., *Kosakonia*, *Lactobacillus*, *Weissella*, *Pediococcus*, *Pantoea*, and *Bacillus*), probably means a clear shift from micro-aerobic conditions to strictly anaerobic conditions in the fermentation system. Previous results concerned with prokaryotic community distribution of PM clearly showed that both Clostridiaceae and methanogens increased their abundances with pit age in the PM (Tao et al., 2014). Like *Clostridiaceae* (within *Clostridiales*) (Tao et al., 2014), some members of *Anaerolineaceae* require syntrophic association with a hydrogenotrophic methanogen for efficient growth and produce acetate, lactate, formate, and hydrogen as end products (Yamada et al., 2007). Given that the syntrophic relationship between *Clostridiaceae* and hydrogenotrophic methanogens maintains the stability of community structure as well as forms flavor constituents during the process (Ding et al., 2015), the physiological characteristics and functions of *Anaerolineaceae* need to be explored further. Moreover, it is striking to find that there were significantly more *Pseudomonadales* taxa present in TCSSF processes than in SCSSF processes. Some members of *Pseudomonadales* such as *Pseudomonas* and *Acinetobacter* have been reported as spoilage-associated bacteria that have negative impacts on fermentation (Wang et al., 2017b). At the beginning of fermentation, genera *Weissella*, *Staphylococcus*, and *Bacillus* were more abundant in TCSSF processes than in SCSSF. According to previous studies (Wang et al., 2017b), variations in the abundance of these taxa might be driven by differences in the microbial community of operating environments (indoor floors, tools, and other unknown environments) or cellars. *Bacillus*, especially *Bacillus licheniformis*, has previously been characterized as the producer of various enzymes, such as amylases, proteases, and lipases, in FG (Gao et al., 2014; Wang et al., 2017a). *Staphylococcus* has been reported as a provider of various enzymes and antibacterial potential (such as soy sauce and Korean soybean paste) (Nam et al., 2012; Yan et al., 2013). However, some members of *Staphylococcus* such as *Staphylococcus aureus* and *Staphylococcus argenteus* are involved in numerous food poisoning outbreaks as an important foodborne pathogen (Wu et al., 2020).

Nonetheless, the bacterial composition was remarkably consistent between SCSSF and TCSSF processes, with only small differences in bacterial diversity and evenness (Shannon) throughout the processes. In contrast, the fungal community in SCSSF processes underwent a more significant changes than that in TCSSF processes during the start-up stage, indicating the beginning of differentiation. Afterward, samples from SCSSF and TCSSF processes were clearly separated in the PCoA ordination space based on fungal community. This might be due to the different decline rates of the relative abundance of *Eurotiales*, which was represented mainly by *Thermoascus* and *Aspergillus*, and the latter has previously been characterized as the major producer of many extracellular enzymes (such as amylase and glucoamylase) that have been associated with the degradation of starch materials (Chen et al., 2014). Most importantly, the relative abundances of *Aspergillus* as well as *Rhizomucor* (within *Mucorales*) were significantly higher in the TCSSF process than in SCSSF (*p* < 0.05), but the amylolytic activity in turn was obviously lower in the TCSSF process than in SCSSF. There is no reliable explanation for this contradiction. Moreover, a clear shift from *Eurotiales* to *Saccharomycetales* was observed throughout the process, suggesting that *Eurotiales* was less tolerant than *Saccharomycetales* to temperature. Pearson’s correlation analyses further clarified this point. After the start-up stage, the fungal community was dominated by the order *Saccharomycetales* with a significant level of *Kazachstania* (19–79%) and *Saccharomyces*. Members of genus *Kazachstania* are typically encountered at low frequency in fermenting grapes (Nisiotou and Nychas, 2008; Bhattacharya et al., 2013) but have not been reported in Chinese Baijiu fermentation. So far, only a few species of this genus have been extensively characterized. Jood et al. (2017) pointed out that some members of *Kazachstania* have positive aroma attributes, but their fermentation power is weaker than that of *Saccharomyces cerevisiae* (Jood et al., 2017). We therefore tentatively speculated that the discrepancy of ethanol contents between SCSSF and TCSSF processes was related to the presence of the genus *Saccharomyces*, which was abundant in the SCSSF process (3–24%) but nearly absent in TCSSF. However, the mixed fermentations of *Kazachstania* spp. and *S. cerevisiae* might lead to the undesirable chemical profile due to excessive levels of higher alcohols that have negative impacts on liquor (Jood et al., 2017).

Moreover, db-RDA results revealed that starch and pH were the most important factors influencing both bacterial and fungal communities at the start-up stage, characterized by increasing microbial diversity and evenness. As expected, there was a strong positive correlation between glucose and bacterial and fungal compositions at the heating stages (days 7–25). Furthermore, Mantel tests showed that changes in starch, moisture, and pH had significant effects on bacterial composition in both SCSSF and TCSSF processes. However, starch and moisture had significant effects only on fungi composition during the TCSSF process. Previous studies also revealed that the performances of microorganisms were associated with complex microbial interactions (Liu et al., 2017; Wang et al., 2019), whereas microbial interactions were associated with microbial succession and diversity (Tan et al., 2019). From a network perspective, TCSSF possessed relatively stable microbiomes compared with SCSSF, which was probably due to the milder physicochemical conditions. Consequent to these findings, we argue that fermentation parameters drove the different microbial compositions and succession rates, which might also be due to slight differences in initial operating parameters.

## Conclusion

The microecology variations observed during the SCSSF and TCSSF processes reveal that fungal composition contributes to a larger extent than bacterial composition to such variations. The microbial communities can accommodate variations in physicochemical conditions by changes in the thermotolerant and acid-resistant community structure during the SSF process. However, different physicochemical variables in turn will affect microbial composition during the SCSSF and TCSSF processes. Understanding the physicochemical changes related to the succession of microbial communities during the SSF of CSFB will ultimately teach people how to harness the power of microbes to obtain better Baijiu products.

## Data Availability Statement

The datasets generated for this study can be found on the NCBI BioProject PRJNA622890.

## Author Contributions

TG and YL contributed to the design of the study and wrote the manuscript. KC organized the database. MO and JZ performed the statistical analysis. All authors contributed to manuscript revision, and read and approved the submitted version.

## Conflict of Interest

JZ was employed by Chengdu Shuzhiyuan of Liquor Co., Ltd. The remaining authors declare that the research was conducted in the absence of any commercial or financial relationships that could be construed as a potential conflict of interest.

## Footnotes

1 http://vegan.r-forge.r-project.org/

## Supplementary Material

FIGURE S1 Sampling design. Every FG sample (▲) was collected from six different positions (the upper layer of pit and the middle layer of pit, 200 g FG at each position), and well mixed as one sample.

FIGURE S2 Rarefaction analysis of the next generation sequencing of the bacterial 16S rRNA **(A)** and fungal ITS1 **(B)** gene from FG based on MEGAN7.

## References

[B1] Bal J. Yun S. H. Yeo S. H. Kim J. M. Kim B. T. Kim D. H. (2017). Effects of initial moisture content of Korean traditional wheat-based fermentation starter nuruk on microbial abundance and diversity. *Appl. Microbiol. Biotechnol.* 101 2093–2106. 10.1007/s00253-016-8042-2 27975136

[B2] Bastian M. Heymann S. Jacomy M. (2009). Gephi: an open source software for exploring and manipulating networks. *ICWSM* 8 361–362. 10.13140/2.1.1341.1520

[B3] Bhattacharya I. Yan S. Yadav J. S. S. Tyagi R. D. Surampalli R. (2013). Saccharomyces unisporus: biotechnological potential and present status. *Compr. Rev. Food Sci. Food Saf.* 12 353–363. 10.1111/1541-4337.12016 33412685

[B4] Bokulich N. A. Collins T. S. Masarweh C. Allen G. Heymann H. Ebeler S. E. (2016). Associations among wine grape microbiome, metabolome, and fermentation behavior suggest microbial contribution to regional wine characteristics. *mBio* 7 e631–e616. 10.1128/mbio.00631-16 27302757 PMC4959672

[B5] Bokulich N. A. Mills D. A. (2013). Facility-specific “House” microbiome drives microbial landscapes of artisan cheesemaking plants. *Appl. Environ. Microb.* 79 5214–5223. 10.1128/AEM.00934-13 23793641 PMC3753952

[B6] Bravo L. Siddhuraju P. Saura-Calixto F. (1998). Effect of various processing methods on the in vitro starch digestibility and resistant starch content of indian pulses. *J. Agr. Food Che.* 46 4667–4674. 10.1021/jf980251f

[B7] Caporaso J. G. Kuczynski J. Stombaugh J. Bittinger K. Bushman F. D. Costello E. K. (2010). QIIME allows analysis of high-throughput community sequencing data. *Nat. Methods.* 7 335–336. 10.1038/nmeth.f.303 20383131 PMC3156573

[B8] Chen B. Wu Q. Xu Y. (2014). Filamentous fungal diversity and community structure associated with the solid state fermentation of Chinese Maotai-flavor liquor. *Int. J. Food Microbiol.* 179 80–84. 10.1016/j.ijfoodmicro.2014.03.011 24742997

[B9] Clarke K. R. (1993). Non-parametric multivariate analyses of changes in community structure. *Aust. J. Ecol.* 18 117–143. 10.1111/j.1442-9993.1993.tb00438.x

[B10] Ding X. F. Wu C. D. Huang J. Zhou R. Q. (2015). Interphase microbial community characteristics in the fermentation cellar of Chinese luzhou-flavor liquor determined by PLFA and DGGE profiles. *Food Res. Int.* 72 16–24. 10.1016/j.foodres.2015.03.018

[B11] Edgar R. C. (2010). Search and clustering orders of magnitude faster than BLAST. *Bioinformatics* 26 2460–2461. 10.1093/bioinformatics/btq461 20709691

[B12] Faust K. Lahti L. Gonze D. de Vos W. M. Raes J. (2015). Metagenomics meets time series analysis: unraveling microbial community dynamics. *Curr. Opin. Microbio.* 25 56–66. 10.1016/j.mib.2015.04.004 26005845

[B13] Gao W. J. Fan W. L. Xu Y. (2014). Characterization of the key odorants in light aroma type chinese liquor by gas chromatography-olfactometry, quantitative measurements, aroma recombination, and omission studies. *J. Agric. Food Chem.* 62 5796–5804. 10.1021/jf501214c 24909925

[B14] Ghanimeh S. A. Saikaly P. E. Li D. El-Fadel M. (2013). Population dynamics during startup of thermophilic anaerobic digesters: the mixing factor. *Waste Manage.* 33 2211–2218. 10.1016/j.wasman.2013.06.011 23830181

[B15] Hu X. L. Du H. Ren C. Xu Y. (2016). Illuminating anaerobic microbial community and cooccurrence patterns across a quality gradient in Chinese liquor fermentation pit muds. *Appl. Environ. Microb.* 82 2506–2515. 10.1128/AEM.03409-15 26896127 PMC4959489

[B16] Huang Y. Kuang Z. Wang W. Cao L. (2016). Exploring potential bacterial and fungal biocontrol agents transmitted from seeds to sprouts of wheat. *Biol. Control.* 98 27–33. 10.1016/j.biocontrol.2016.02.013

[B17] Jiang Z. M. Chen Y. Wang D. L. Wang X. L. Shang K. Zhang S. (2016). The influence of porosity of the grain medium on the flavour formation of Chinese liquor during solid-state fermentation. *J. I. Brewing.* 122 468–472. 10.1002/jib.345

[B18] Jin G. Y. Zhu Y. Xu Y. (2017). Mystery behind Chinese liquor fermentation. *Trends Food Sci. Tech.* 63 18–28. 10.1016/j.tifs.2017.02.016

[B19] Jood I. Hoff J. W. Setati M. E. (2017). Evaluating fermentation characteristics of Kazachstania spp. and their potential influence on wine quality. *World J. Microb. Bio.* 33:129. 10.1007/s11274-017-2299-1 28585169

[B20] Jung J. Y. Lee S. H. Lee H. J. Seo H. Y. Park W. S. Jeon C. O. (2012). Effects of *Leuconostoc mesenteroides* starter cultures on microbial communities and metabolites during kimchi fermentation. *Int. J. Food Microbiol.* 153 378–387. 10.1016/j.ijfoodmicro.2011.11.030 22189023

[B21] Li S. Li P. Liu X. Luo L. Lin W. (2016). Bacterial dynamics and metabolite changes in solid-state acetic acid fermentation of Shanxi aged vinegar. *Appl. Microbiol. Biot.* 100 4395–4411. 10.1007/s00253-016-7284-3 26754813

[B22] Li P. Lin W. Liu X. Wang X. Luo L. (2016). Environmental factors affecting microbiota dynamics during traditional solid-state fermentation of Chinese daqu starter. *Front. Microbiol.* 7:1237. 10.3389/fmicb.2016.01237 27540378 PMC4972817

[B23] Liu Y. Rousseaux S. Tourdot-Marechal R. Sadoudi M. Gougeon R. Schmitt-Kopplin P. (2017). Wine microbiome: a dynamic world of microbial interactions. *Crit. Rev. Food Sci.* 57 856–873. 10.1080/10408398.2014.983591 26066835

[B24] Lu H. Z. Ca Y. M. Wu Z. W. Jia J. H. Bai F. Y. (2004). Kazachstania aerobia sp nov., an ascomycetous yeast species from aerobically deteriorating corn silage. *Int. J. Syst. Evol. Micr.* 54 2431–2435. 10.1099/ijs.0.63257-0 15545492

[B25] Miller G. L. (1959). Use of dinitrosalicylic acid reagent for determination of reducing sugar. *Anal. Chem.* 31 426–428. 10.1021/ac60147a030

[B26] Nam Y. D. Lee S. Y. Lim S. I. (2012). Microbial community analysis of Korean soybean pastes by next-generation sequencing. *Int. J. Food Microbiol.* 155 36–42. 10.1016/j.ijfoodmicro.2012.01.013 22305887

[B27] Nisiotou A. A. Nychas G. E. (2008). Kazachstania hellenica sp. nov., a novel ascomycetous yeast from a Botrytis-affected grape must fermentation. *Int. J. Syst. Evol. Microbiol.* 58 1263–1267. 10.1099/ijs.0.65649-0 18450725

[B28] Smid E. J. Hugenholtz J. (2010). Functional genomics for food fermentation processes. *Annu. Rev. Food Sci. T.* 1 497–519. 10.1146/annurev.food.102308.124143 22129346

[B29] Smid E. J. Lacroix C. (2013). Microbe-microbe interactions in mixed culture food fermentations. *Curr. Opin. Biotech.* 24 148–154. 10.1016/j.copbio.2012.11.007 23228389

[B30] Sogin M. L. Morrison H. G. Huber J. A. Welch D. M. Huse S. M. Neal P. R. (2006). Microbial diversity in the deep sea and the underexplored ‘rare 15 biosphere’. *Proc. Natl. Acad. Sci. U.S.A.* 103 12115–12120. 10.1073/pnas.0605127103 16880384 PMC1524930

[B31] Sun W. N. Xiao H. Z. Peng Q. Zhang Q. G. Li X. X. Han Y. (2016). Analysis of bacterial diversity of Chinese luzhou-flavor liquor brewed in different seasons by Illumina Miseq sequencing. *Ann. Microbiol.* 66 1293–1301. 10.1007/s13213-016-1223-5

[B32] Tan Y. W. Zhong H. P. Zhao D. Du H. Xu Y. (2019). Succession rate of microbial community causes flavor difference in strong-aroma Baijiu making process. *Int. J. Food Microbio.* 311:108350. 10.1016/j.ijfoodmicro.2019.108350 31614280

[B33] Tang Y. Q. Ji P. Hayashi J. Koike Y. Wu X. L. Kida K. (2011). Characteristic microbial community of a dry thermophilic methanogenic digester: its longterm stability and change with feeding. *Appl. Microbiol. Biotechnol.* 91 1447–1461. 10.1007/s00253-011-3479-9 21789494

[B34] Tao Y. Li J. B. Rui J. P. Xu Z. C. Zhou Y. Hu X. H. (2014). Prokaryotic communities in pit mud from different-aged cellars used for the production of Chinese strong-flavored liquor. *Appl. Environ. Microb.* 88 2254–2260. 10.1128/AEM.04070-13 24487528 PMC3993157

[B35] Troy S. M. Nolan T. Kwapinski W. Leahy J. J. Healy M. G. Lawlor P. G. (2012). Effect of sawdust addition on composting of separated raw and anaerobically digested pig manure. *J. Environ. Manage.* 111 70–77. 10.1016/j.jenvman.2012.06.035 22824375

[B36] Vázquez B. Y. Pirrung M. Gonzalez A. Knight R. (2013). EMPeror: a tool for visualizing high-throughput microbial community data. *GigaScience* 2:16. 10.1186/2047-217X-2-16 24280061 PMC4076506

[B37] Vermeulen A. Daelman J. Van Steenkiste J. Devlieghere F. (2012). Screening of different stress fa.ctors and development of growth/no growth models for Zygosaccharomyces rouxii in modified Sabouraud medium, mimicking intermediate moisture foods (IMF). *Food Microbiol.* 32 389–396. 10.1016/j.fm.2012.07.019 22986205

[B38] Wang S. Wu Q. Nie Y. Wu J. Xu Y. (2019). Construction of synthetic microbiota for reproducible flavor metabolism in Chinese light aroma type liquor produced by solid-state fermentation. *Appl. Microbiol. Biotechnol.* 10:e3090-18. 10.1128/AEM.03090-18 30850432 PMC6498162

[B39] Wang X. S. Du H. Xu Y. (2017a). Source tracking of prokaryotic communities in fermented grain of Chinese Strong-flavor liquor. *Int. J. Food Microbio.* 244 27–45. 10.1016/j.ijfoodmicro.2016.12.018 28064120

[B40] Wang X. S. Du H. Zhang Y. Xu Y. (2017b). Environmental microbiota drives microbial succession and metabolic profiles during Chinese liquor fermentation. *Appl. Environ. Microb.* 84:e2369-17. 10.1128/AEM.02369-17 29196296 PMC5795089

[B41] Wu S. Huang J. H. Zhang F. Dai J. S. Pang R. Zhang J. M. (2020). *Staphylococcus argenteus* isolated from retail foods in China: incidence, antibiotic resistance, biofilm formation and toxin gene profile. *Food Microbiol.* 91:103531. 10.1016/j.fm.2020.103531 32539963

[B42] Xiao C. Lu Z. M. Zhang X. J. Wang S. T. Ao L. Shen C. H. (2017). Bio-Heat is a key environmental eriver shaping the microbial community of medium-temperature daqu. *Appl. Environ. Microb.* 83 e1550–e1517. 10.1128/AEM.01550-17 PMC5691423 28970223

[B43] Yamada T. Imachi H. Ohashi A. Harada H. Hanada S. Kamagata Y. (2007). Bellilinea caldifistulae gen. nov., sp nov and Longilinea arvoryzae gen. nov., sp nov., strictly anaerobic, filamentous bacteria of the phylum Chloroflexi isolated from methanogenic propionate-degrading consortia. *Int. J. Syst. Evol. Micr.* 57 2299–2306. 10.1099/ijs.0.65098-0 17911301

[B44] Yan Y. Z. Qian Y. L. Feng D. J. Chen J. Y. Han B. Z. (2013). Microbial composition during Chinese soy sauce koji-making based on culture dependent and independent methods. *Food Microbiol.* 34 189–195. 10.1016/j.fm.2012.12.009 23498197

[B45] Zhang J. Kobert K. Flouri T. Stamatakis A. (2014). PEAR: a fast and accurate illumina paired-end reAd mergeR. *Bioinformatics* 30 614–620. 10.1093/bioinformatics/btt593 24142950 PMC3933873

[B46] Zhang L. Che Z. M. Xu W. Z. Yue P. Li R. Li Y. F. (2020). Dynamics of physicochemical factors and microbial communities during ripening fermentation of Pixian Doubanjiang, a typical condiment in Chinese cuisine. *Food Microbiol.* 86:103342. 10.1016/j.fm.2019.103342 31703884

[B47] Zou W. Ye G. B. Zhang K. Z. (2018). Diversity, function, and application of clostridium in Chinese strong flavor baijiu ecosystem: a review. *J. Food Sci.* 83 1193–1199. 10.1111/1750-3841.14134 29660763

