# Supplementary figures and images for: Physicochemical Factors Affecting Microbiota Dynamics During Traditional Solid-State Fermentation of Chinese Strong-Flavor Baijiu
Source: Front Microbiol. 2020 Sep 9;11:2090. doi: 10.3389/fmicb.2020.02090 (PMC7509048; doi:10.3389/fmicb.2020.02090)

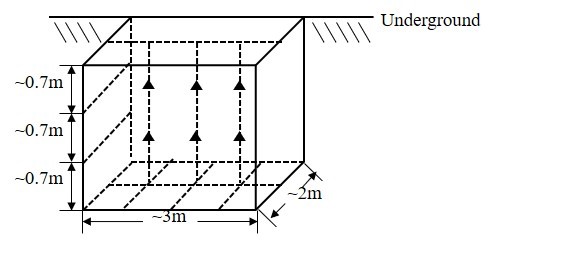

Supplement: FIGURE S1 — Sampling design. Every FG sample (▲) was collected from six different positions (the upper layer of pit and the middle layer of pit, 200 g FG at each position), and well mixed as one sample. [file Image_1.JPEG]

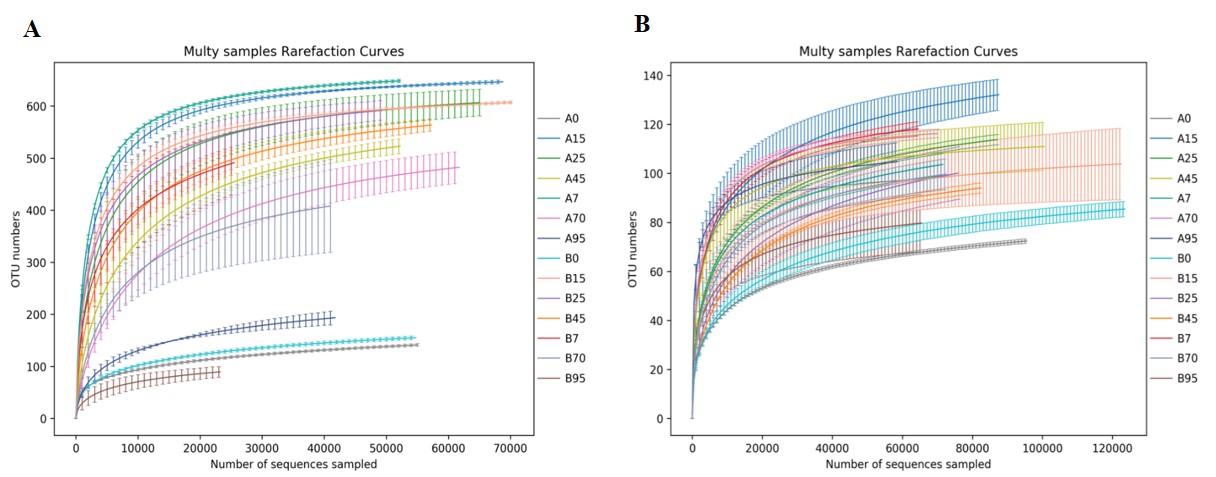

Supplement: FIGURE S2 — Rarefaction analysis of the next generation sequencing of the bacterial 16S rRNA (A) and fungal ITS1 (B) gene from FG based on MEGAN7. [file Image_2.JPEG]
