# Supplementary material for: Physicochemical Factors Affecting Microbiota Dynamics During Traditional Solid-State Fermentation of Chinese Strong-Flavor Baijiu
Source: Front Microbiol. 2020 Sep 9;11:2090. doi: 10.3389/fmicb.2020.02090 (PMC7509048; doi:10.3389/fmicb.2020.02090)
Supplement: Supplementary file 3 [file Table_1.DOCX]

**TABLE S1 | Relative abundance of bacterial 16S rRNA and fungal ITS genes of FG at the genus level (average relative abundance > 1%).**

| Order | Genus | Relative abundance (%) | | | | | | | | | | | | | |  |
| --- | --- | --- | --- | --- | --- | --- | --- | --- | --- | --- | --- | --- | --- | --- | --- | --- |
|  |  | S0 | S7 | S15 | S25 | S45 | S70 | S95 | T0 | T7 | T15 | T25 | T45 | T70 | T95 | |
| *Lactobacillales* | *Lactobacillus* | 17.37 ± 9.80^d^ | 5.09 ± 0.92^d^ | 26.35 ± 10.64^cd^ | 45.73 ± 27.57^bc^ | 76.88 ± 8.38^a^ | 87.82 ± 5.03^a^ | 86.52 ± 5.44^a^ | 12.67 ± 5.24^d^ | 1.08 ± 0.79^d^ | 11.14 ± 14.14^d^ | 24.56 ± 38.34^cd^ | 58.64 ± 11.78^ab^ | 81.32 ± 4.76^a^ | 85.26 ± 21.35^a^ | |
|  | *Weissella* | 12.05 ± 1.71^b^ | 1.39 ± 0.46^c^ | 2.12 ± 2.38^c^ | 0.36 ± 0.24^c^ | 0.08 ± 0.04^c^ | 0.23 ± 0.20^c^ | 0.30 ± 0.13^c^ | 17.40 ± 6.26^a^ | 0.54 ± 0.75^c^ | 0.77 ± 0.47^c^ | 0.80 ± 0.54^c^ | 0.13 ± 0.04^c^ | 0.15 ± 0.03^c^ | 0.05 ± 0.05^c^ | |
|  | *Pediococcus* | 11.24 ± 2.05^a^ | 2.20 ± 0.5^b^ | 1.14 ± 0.21^b^ | 0.57 ± 0.54^b^ | 0.05 ± 0.01^b^ | 0.10 ± 0.01^b^ | 0.02 ± 0.01^b^ | 10.28 ± 4.31^a^ | 0.97 ± 1.55^b^ | 1.40 ± 1.04^b^ | 1.35 ± 0.47^b^ | 0.10 ± 0.01^b^ | 0.14 ± 0.00^b^ | 0.05 ± 0.01^b^ | |
| *Enterobacterales* | *Kosakonia* | 21.71 ± 3.65^a^ | 1.18 ± 0.37^b^ | 0.99 ± 0.30^b^ | 0.49 ± 0.13^b^ | 0.14 ± 0.14^b^ | 0.70 ± 0.09^b^ | 0.02 ± 0.01^b^ | 21.14 ± 3.62^a^ | 0.60 ± 0.85^b^ | 0.65 ± 0.39^b^ | 0.75 ± 0.23^b^ | 0.05 ± 0.01^b^ | 0.16 ± 0.02^b^ | 0.07 ± 0.01^b^ | |
|  | *Pantoea* | 11.31 ± 5.13^a^ | 0.27 ± 0.01^c^ | 0.27 ± 0.01^c^ | 0.13 ± 0.01^c^ | 0.07 ± 0.01^c^ | 0.19 ± 0.24^c^ | 0.01 ± 0.01^c^ | 3.66 ± 0.53^b^ | 0.16 ± 0.02^c^ | 0.21 ± 0.02^c^ | 0.19 ± 0.01^c^ | 0.01 ± 0.01^c^ | 0.06 ± 0.01^c^ | 0.05 ± 0.01^c^ | |
| *Bacillales* | *Bacillus* | 6.94 ± 1.81^b^ | 2.55 ± 0.52^c^ | 2.29 ± 0.83^c^ | 1.63 ± 0.81^c^ | 0.52 ± 0.42^cd^ | 0.70 ± 0.12^cd^ | 0.40 ± 2.77^d^ | 13.71 ± 0.10^a^ | 0.85 ± 0.06^cd^ | 1.22 ± 0.07^c^ | 1.32 ± 0.07^c^ | 0.33 ± 0.01^d^ | 0.31 ± 0.01^d^ | 0.20 ± 0.18^d^ | |
|  | *Staphylococcus* | 5.95 ± 2.67^b^ | 0.77 ± 0.39^c^ | 0.72 ± 0.11^c^ | 0.58 ± 0.32^c^ | 0.11 ± 0.14^c^ | 0.20 ± 0.11^c^ | 1.90 ± 1.25^c^ | 8.76 ± 4.18^a^ | 0.40 ± 0.40^c^ | 0.48 ± 0.60^c^ | 0.58 ± 0.28^c^ | 0.06 ± 0.46^c^ | 0.13 ± 0.01^c^ | 0.12 ± 0.01^c^ | |
| *Bacteroidales* | *Proteiniphilum* | 0.00 ± 0.00^e^ | 4.78 ± 0.39^a^ | 4.06 ± 0.52^b^ | 2.43 ± 0.98^c^ | 0.92 ± 0.51^d^ | 0.32 ± 0.01^d^ | 0.27 ± 0.42^d^ | 0.00 ± 0.00^e^ | 0.59 ± 0.49^d^ | 0.93 ± 0.11^d^ | 0.70 ± 0.34^d^ | 0.43 ± 0.13^d^ | 0.14 ± 0.00^d^ | 0.00 ± 0.00^e^ | |
|  | *Bacteroides* | 0.04 ± 0.00^b^ | 1.13 ± 0.01^ab^ | 1.34 ± 0.63^ab^ | 0.37 ± 0.19^ab^ | 0.29 ± 0.22^ab^ | 0.13 ± 0.01^ab^ | 0.23 ± 0.23^ab^ | 0.04 ± 0.00^ab^ | 0.20 ± 0.17^ab^ | 0.65 ± 0.21^ab^ | 1.24 ± 0.79^ab^ | 0.36 ± 0.01^ab^ | 0.54 ± 0.30^ab^ | 2.05 ± 3.37^a^ | |
|  | *Fermentimonas* | 0.00 ± 0.00^d^ | 1.92 ± 0.01^a^ | 1.87 ± 0.20^a^ | 1.04 ± 0.49^b^ | 0.40 ± 0.18^c^ | 0.16 ± 0.00^cd^ | 0.00 ± 0.00^d^ | 0.00 ± 0.00^d^ | 0.33 ± 0.29^cd^ | 0.45 ± 0.01^cd^ | 0.33 ± 0.19^cd^ | 0.19 ± 0.01^d^ | 0.04 ± 0.00^d^ | 0.00 ± 0.00^d^ | |
| *Pseudomonadales* | *Acinetobacter* | 0.86 ± 0.36^d^ | 2.99 ± 0.45^c^ | 2.65 ± 0.59^d^ | 1.59 ± 0.86^d^ | 1.18 ± 0.67^d^ | 0.35 ± 0.10^d^ | 0.17 ± 0.12^d^ | 0.82 ± 0.12^d^ | 5.12 ± 0.21^a^ | 6.91 ± 3.37^a^ | 4.51 ± 0.84^b^ | 3.12 ± 0.33^bc^ | 0.78 ± 0.38^d^ | 1.03 ± 1.70^d^ | |
|  | *Pseudomonas* | 0.05 ± 0.00^c^ | 0.81 ± 0.11^bc^ | 1.05 ± 0.23^bc^ | 0.61 ± 0.44^bc^ | 0.33 ± 0.18^c^ | 0.10 ± 0.00^c^ | 0.08 ± 0.01^c^ | 0.06 ± 0.01^c^ | 3.38 ± 1.19^a^ | 3.12 ± 0.99^a^ | 2.52 ± 1.13^a^ | 1.40 ± 0.22^b^ | 0.43 ± 0.28^bc^ | 0.03 ± 0.00^d^ | |
| *Rhodospirillales* | *Acetobacter* | 1.71 ± 1.38^b^ | 4.25 ± 1.54^a^ | 0.30 ± 0.01^c^ | 0.37 ± 0.02^c^ | 0.09 ± 0.01^c^ | 0.07 ± 0.00^c^ | 0.02 ± 0.00^c^ | 2.37 ± 0.40^b^ | 0.12 ± 0.01^c^ | 0.24 ± 0.02^c^ | 0.27 ± 0.01^c^ | 0.03 ± 0.00^c^ | 0.03 ± 0.00^c^ | 0.00 ± 0.00^c^ | |
| *Anaerolinaeles* | *Longilinea* | 0.00 ± 0.00^d^ | 2.55 ± 0.17^ab^ | 1.65 ± 0.14^bc^ | 1.50 ± 0.85^cd^ | 0.55 ± 0.32^cd^ | 0.16 ± 0.04^d^ | 0.00 ± 0.00^d^ | 0.00 ± 0.00^d^ | 3.20 ± 2.64^a^ | 2.94 ± 0.34^a^ | 1.83 ± 1.00^ab^ | 1.00 ± 0.45^bc^ | 0.37 ± 0.23^cd^ | 0.00 ± 0.00^d^ | |
| *Methylococcales* | *Methylobacter* | 0.00 ± 0.00^e^ | 2.84 ± 0.18^ab^ | 2.04 ± 0.38^bc^ | 1.43 ± 0.09^cd^ | 0.63 ± 0.03^d^ | 0.35 ± 0.02^e^ | 0.01 ± 0.00^e^ | 0.00 ± 0.00^e^ | 3.71 ± 3.08^a^ | 3.49 ± 0.89^a^ | 3.34 ± 1.94^a^ | 2.11 ± 0.09^bc^ | 0.90 ± 0.04^cd^ | 0.01 ± 0.00^e^ | |
| *Myxococcales* | *Anaeromyxobacter* | 0.00 ± 0.11^d^ | 1.74 ± 0.19^a^ | 1.18 ± 0.51^b^ | 1.10 ± 0.02^b^ | 0.32 ± 0.15^cd^ | 0.14 ± 0.00^d^ | 0.00 ± 0.00^d^ | 0.00 ± 0.00^d^ | 0.63 ± 0.49^cd^ | 0.77 ± 0.15^c^ | 0.65 ± 0.26^d^ | 0.43 ± 0.11^d^ | 0.23 ± 0.28^d^ | 0.00 ± 0.00^d^ | |
| *Clostridiales* | *Saccharofermentans* | 0.00 ± 0.01^d^ | 0.57 ± 0.02^bc^ | 0.33 ± 0.11^c^ | 0.34 ± 0.04^c^ | 0.16 ± 0.02^cd^ | 0.07 ± 0.00^d^ | 0.02 ± 0.02^d^ | 0.00 ±0.00^d^ | 1.14 ± 0.24^a^ | 0.81 ± 0.15^b^ | 1.00 ± 0.35^a^ | 0.37 ± 0.12^bc^ | 0.36 ± 0.02^d^ | 0.00 ± 0.00^d^ | |
|  | *Clostridium* | 0.00 ± 0.00^d^ | 0.51 ± 0.12^bc^ | 0.76 ± 0.21^b^ | 0.62 ± 0.22^bc^ | 0.28 ± 0.11^c^ | 0.27 ±0.02^cd^ | 0.02 ± 0.01^d^ | 0.00 ± 0.00^d^ | 1.09 ± 0.34^a^ | 0.82 ± 0.24^b^ | 0.83 ± 0.44^b^ | 0.33 ± 0.04^c^ | 0.22 ± 0.0.3^cd^ | 0.00 ± 0.00^d^ | |
| *Flavobacteriales* | *Flavobacterium* | 0.00 ± 0.00^c^ | 0.11 ± 0.02^c^ | 0.20 ± 0.04^bc^ | 1.48 ± 0.25^a^ | 0.42 ± 0.16^b^ | 0.02 ± 0.01^c^ | 0.10 ± 0.01^c^ | 0.00 ± 0.00^c^ | 0.05 ± 0.01^c^ | 0.34 ± 0.13^b^ | 1.19 ± 0.54^a^ | 0.23 ± 0.05^bc^ | 0.04 ± 0.02^c^ | 0.00 ± 0.00^c^ | |
| Others |  | 10.77 | 62.35 | 48.69 | 37.63 | 16.58 | 7.92 | 9.91 | 9.09 | 75.84 | 62.66 | 52.04 | 30.68 | 13.65 | 11.08 | |
| *Saccharomycetales* | *Kazachstania* | 0.02 ± 0.00^e^ | 74.16 ± 4.38^ab^ | 64.18 ± 16.55^abc^ | 65.44 ± 6.34^abc^ | 51.34 ± 3.10^cd^ | 47.50 ± 1.30^d^ | 22.92 ± 0.00^e^ | 0.02 ± 0.00^e^ | 20.36 ± 11.08^e^ | 57.14 ± 1.91^bcd^ | 79.65 ± 7.80^a^ | 79.71 ± 4.50^a^ | 70.36 ± 19.15^a^ | 68.87 ± 15.14^abc^ | |
|  | *Saccharomyces* | 0.00 ± 0.00^d^ | 5.13 ± 1.24^cd^ | 6.78 ± 1.61^c^ | 13.46 ± 4.05^b^ | 23.82 ± 7.12^a^ | 15.74 ± 4.87b | 3.65 ± 2.93^cd^ | 0.00 ± 0.00^d^ | 0.06 ± 0.01^d^ | 0.07 ± 0.02^d^ | 0.41 ± 0.55^d^ | 0.32 ± 0.12^d^ | 0.38 ± 0.47^d^ | 0.99 ± 0.24^d^ | |
|  | *Torulaspora* | 0.00 ± 0.00^d^ | 2.53 ± 0.12^b^ | 2.58 ±0.08^b^ | 5.22 ± 1.25^a^ | 1.10 ± 0.20^bc^ | 0.84 ± 0.03^c^ | 0.14 ± 0.01^cd^ | 0.00 ± 0.00^d^ | 0.07 ± 0.01^d^ | 0.09 ± 0.01^d^ | 0.05 ± 0.02^d^ | 0.02 ± 0.01^d^ | 0.01 ± 0.00^d^ | 0.00 ± 0.00^d^ | |
|  | *Pichia* | 0.01 ± 0.01^c^ | 0.24 ± 0.05^bc^ | 0.20 ± 0.03^bc^ | 0.16 ± 0.01^c^ | 0.36 ± 0.06^bc^ | 0.79 ± 0.21^b^ | 0.03 ± 0.01^c^ | 0.01 ± 0.00^c^ | 1.17 ± 0.56^ab^ | 0.84 ± 0.42^b^ | 0.22 ± 0.02^bc^ | 0.85 ± 0.23^b^ | 1.46 ± 0.52^a^ | 0.08 ± 0.01^c^ | |
| *Eurotiales* | *Thermoascus* | 38.35 ± 10.18^a^ | 5 .03 ± 1.77^de^ | 3.53 ± 7.71^de^ | 4.77± 2.73^de^ | 3.70 ± 4.14^de^ | 11.36 ± 2.10^cd^ | 0.70 ± 0.46^e^ | 29.79 ± 5.85^b^ | 26.04 ± 2.80^b^ | 15.52 ± 3.12^c^ | 8.69 ± 4.78^bcd^ | 3.18 ± 0.65^e^ | 5.77 ±4.43^de^ | 4.74 ± 4.08^de^ | |
|  | *Aspergillus* | 22.55 ± 2.40^c^ | 2.17 ± 0.89^f^ | 1.82 ± 1.00^f^ | 1.40 ± 0.34^f^ | 1.62 ± 1.27^f^ | 3.19 ± 0.45^f^ | 6.43 ± 3.51^ef^ | 41.67 ± 9.19^a^ | 33.14 ± 3.82^b^ | 13.42 ± 0.58^d^ | 7.37 ± 2.96^ef^ | 5.16 ± 2.09^ef^ | 6.66 ± 3.64^ef^ | 10.32 ± 3.58^de^ | |
|  | *Thermomyces* | 0.45 ± 0.05^b^ | 1.57 ± 1.76^b^ | 0.21 ± 0.25^b^ | 0.11 ± 0.08^b^ | 0.08 ± 0.04^b^ | 0.08 ± 0.06^b^ | 13.07 ± 7.79^a^ | 1.10 ± 1.45^b^ | 1.93 ± 2.62^b^ | 0.65 ± 0.78^b^ | 0.09 ± 0.06^b^ | 0.03 ± 0.02^b^ | 0.04 ± 0.06^b^ | 0.04 ± 0.04^b^ | |
|  | *Pseudeurotium* | 0.00 ± 0.00^c^ | 0.06 ± 0.01^c^ | 0.04 ±0.00^c^ | 0.09 ± 0.04^c^ | 0.79 ± 0.50^c^ | 0.27 ± 0.14^c^ | 0.05 ± 0.05^c^ | 0.00 ± 0.00^c^ | 0.45 ± 0.70^c^ | 0.07 ± 0.05^c^ | 0.02 ± 0.02^c^ | 8.58 ± 1.37^a^ | 5.17 ± 5.90^b^ | 0.00 ± 0.00^c^ | |
| *Mucorales* | *Rhizomucor* | 2.28 ± 0.23^b^ | 0.02 ± 0.00^d^ | 0.03 ± 0.01^d^ | 0.02 ± 0.00^d^ | 0.05 ± 0.02^d^ | 0.52 ± 0.21^cd^ | 0.16 ± 0.05^cd^ | 6.17 ± 0.24^a^ | 1.65 ± 0.14^bc^ | 0.33 ± 0.02^d^ | 0.15 ± 0.03^d^ | 0.03 ± 0.00^d^ | 0.28 ± 0.01^d^ | 0.34 ± 0.02^d^ | |
| [*Trichosporonales*](https://www.ncbi.nlm.nih.gov/Taxonomy/Browser/wwwtax.cgi?mode=Undef&id=1851469&lvl=3&p=has_linkout&p=blast_url&p=genome_blast&lin=f&unlock) | *Apiotrichum* | 0.00 ± 0.00^c^ | 0.12 ± 0.01^c^ | 0.05 ± 0.02^c^ | 1.25 ± 0.45^b^ | 1.47 ± 0.12^b^ | 1.22 ± 0.21^b^ | 4.81 ± 2.45^a^ | 0.00 ± 0.00^c^ | 0.39 ± 0.23^c^ | 0.11 ± 0.02^c^ | 0.08 ± 0.02^c^ | 0.04 ± 0.01^c^ | 0.13 ± 0.02^c^ | 0.01 ± 0.00^c^ | |
| Others |  | 36.34 | 14.00 | 20.58 | 8.08 | 15.67 | 18.49 | 48.04 | 21.24 | 14.74 | 11.76 | 3.27 | 2.08 | 9.74 | 14.61 | |

All data are presented as means ± standard deviations (n=3). Values with different letters in a row mean significant differences at *P* < 0.05 (with false discovery rate correction) as determined by one-way ANOVA Duncan's test.
